# Supplementary material for: Rickettsia felis DNA recovered from a child who lived in southern Africa 2000 years ago
Source: Commun Biol. 2023 Mar 3;6:240. doi: 10.1038/s42003-023-04582-y (PMC9984395; doi:10.1038/s42003-023-04582-y)
Supplement: Supplementary file 2 — Supplementary Information [file 42003_2023_4582_MOESM2_ESM.pdf]

Rickettsia felis DNA recovered from a child who lived in southern Africa 2,000 years ago

Supplementary Notes 1-7

Supplementary Note 1 Skeletal provenience of the boy from Ballito Bay

Ballito Boy (BBayA) was recovered from an archaeological context along the KwaZulu-Natal Province coastline and AMS radiocarbon-dated to 1,980 ± 20 cal. BP (1936 - 1831 cal. BP at 95% probability), *i.e.*, *c*. 2,000 years ago (Schlebusch et al., 2017). The remains were excavated by Schoute-Vanneck and Walsh during the 1960s, first curated at the Durban Museum, and then transferred to the KwaZulu-Natal Museum where it is now curated (accession no. 2009/007). The site from which it was retrieved is said to have been a mound formed by a shell-midden overlooking the beach, about 46 m from the high-water mark. The skeletal material cannot be directly associated with archaeological material from the site as clear stratigraphic context is unknown. Admixture analyses indicate that BBayA cluster with modern Southern San populations (Schlebusch et al., 2017). On account of the high genome coverage (~13-fold) of BBayA, Schlebusch et al. (2017) recalculated the genetic time depth for *Homo sapiens* to between 350 kya and 260 kya. This revised split-estimate coincides with the fossil material from Morocco, dated to *c*. 300 kya (Hublin et al., 2017) and which is viewed as anatomically-transitional between archaic and modern *H. sapiens* (Lombard et al., 2018).

Supplementary Note 2 Immune adaptations of African hunter-gatherers

Of the ~2,100 species of microbes that interact directly with humans (Wardeh et al., 2015), at least 1,415 species are known to be pathogenic, including various bacteria, viruses, fungi, protozoa and helminths (Taylor et al., 2001; Woolhouse and Gowtage-Sequeria, 2005). Approximately 65% of these are zoonotic (Lloyd-Smith et al., 2009) and ~8% are suspected to cause emerging infectious diseases (Dutour, 2013). At least 20 of these pathogens have certain to probable African origin, including hepatitis B, measles, cholera, dengue fever, *P. falciparum* malaria and leishmaniasis, plague and smallpox (Houldcroft and Underdown, 2016; Wolfe et al., 2007). Despite the fact pathogens have long exerted a significant influence on hominin longevity (Rifkin et al., 2017) and human genetic diversity (Pittman et al., 2016), and given that diseases continue to shape our history (Andam et al., 2016), their influence on the biological and socio-cultural evolution of our species, in Africa, is routinely overlooked.

Persistent exposure to pathogens exerted selective pressure on human health (Owers et al., 2017), immune responses (Nédélec et al., 2016), cognitive development (Kessler et al., 2017) and social behaviour (Thornhill and Fincher, 2014). The bio-geographic distribution of *Plasmodium falciparum* (Tanabe et al., 2010) and *Helicobacter pylori* (Linz et al., 2007) exhibits declining genetic diversity, with increasing distance from Africa, with ‘Out of Africa’ estimates of about 58 kyr and 80 kyr ago,

respectively. Indeed, and given that the *H. pylori* association with humans is at least 100,000 years old (Moodley et al., 2012), the current population structure of *H. pylori* may be regarded as mirroring past human expansions and migrations. In addition to *Plasmodium falciparum* (Tanabe et al., 2010), roughly 250 *Plasmodium* species, including *P. vivax*, *P. malariae*, *P. falciparum* and *P. ovale* are highly anthropophilic (Ollomo et al., 2009). Mitochondrial mtDNA analyses confirm that early forms of *P. falciparum* were present by at least 100 kya (Kwiatkowski, 2005; Silva et al., 2011). Some of the first examples of natural selection acting on the human genome involve genetic mutations that confer resistance to malaria. The Duffy negativity locus evolved some 100 ka (Ferwerda et al., 2007) to ~ 60 kya (McManus et al., 2017) and confers resistance against *P. vivax* malaria to many sub-Saharan Africans (Howes et al., 2011). That these and several other malaria-resistant alleles evolved independently (Ko et al., 2011) suggests that malaria exerted a significant degree of selective pressure in prehistory.

In addition to the fact that it appears that persistent exposure to pathogens exerted selective pressure on human immune-related genes (Nédélec et al., 2016; Owers et al., 2017), the antiquity of genetic disease prevention mechanisms, such as the origin of immune-regulating Sia-recognising Ig-like lectin (SIGLEC) genes before 70 kya (Wang et al., 2012), confirms that pathogens played an essential role in human evolution in Africa. More recently, Lopez et al. (2019) has detected strong polygenic adaptation signals for functions related to mast-cell responses to allergens and microbes, and host interactions with viruses also support a history of pathogen-driven selection in the rainforest. In the case of BBayA, the incidence of genomic variants relating to pathogen exposure (Schlebusch et al., 2017) is of particular interest. The FY*A allele, which has a protective effect against malaria, was identified in BBayA, which also carries the ATP2B4 gene variant, another polymorphism which protect against childhood malaria and which appears to have emerged ~ 60 kya (McManus et al., 2017). BBayA does not carry the Duffy null allele, which has a protective effect against *P. vivax* associated malaria. Similarly, the APOL1 gene variant, which confers resistance to African sleeping sickness, is also absent in BBayA.

Supplementary Note 3 *Rickettsia felis* strain LSU-Lb

Rickettsia felis str. LSU-Lb is an obligate mutualist of the parthenogenic booklouse *Liposcelis bostrychophila* (Insecta: Psocoptera), an insect only recently recognized as a host for *R. felis* (Thepparit et al., 2011). *Rickettsia felis* str. LSU-Lb was first isolated in 2010, in Los Angeles County, California, USA. Phylogenomic analysis suggests that *R. felis* str. LSU-Lb diverged from the flea-associated strains. It is suggested that the shared microhabitat between fleas (*e.g.*, cat fleas, *Ctenocephalides felis*) and *L. bostrychophila* and the phoretic relationship of *R. felis*-infected *L. bostrychophila* with vertebrate hosts facilitates the horizontal transmission of *R. felis* from fleas to *L. bostrychophila*.

Supplementary Note 4 Bacterial aDNA damage patterns

Following DNA extraction, the sequencing output, *i.e.*, 'read-counts', is dependent on the sequencing depth of each sequencing run and the presence of sufficient un-damaged DNA strands to detect during sequencing, the latter factor which is, in turn, dependent on the morphology (*i.e.*, the cell wall structure, spore formation, the presence of mycolic acids and guanine-cytosine (GC) content) of different types of pathogenic microbes (Mann et al., 2018). As per Donoghue et al. (2017), mycobacterial aDNA is generally more resistant to degradation compared to mammalian host aDNA, due to the protective presence of the bacterial cell wall and the higher proportion of guanidine and cytosine in the DNA. However, Mann et al. (2018) found that fragmentation patterns within dental calculus are associated with the genomic source of the DNA (human *vs*. microbial) but not with cellular structure (*e.g.*, microbial cell wall type or presence of a surface-layer). Accordingly, it appears that short DNA fragments from taxa with lower GC content genomes should be expected to be more susceptible to loss through denaturation because their melting point for a given fragment length will be lower, and this may contribute significantly to taxonomic misalignments and misidentifications. Consistent with this hypothesis, Mann et al. (2018) found that high GC-content genera had slightly shorter median fragment lengths overall, which accords with a higher retention of short DNA fragments.

Supplementary Note 5 The emergence of a MRCA for the southern African *R. felis* group

Given a lack or temporal signals in the ancient DNA dataset, we were not able to determine chronometric stages in the evolution of the BBayA *R. felis*, nor could we ascertain the emergence of a most recent common ancestor (MRCA) for the southern African *R. felis* group. Molecular clock and divergence analysis of BBayA *R. felis* was performed by using the codon alignment in BEAST v2.5.0 (Bouckaert et al., 2019). A coalescent constant prior and strict molecular clock was used for the Markov chain Monte Carlo (MCMC) chain analysis. Five different runs of 100 million MCMC were performed and sampled every 5,000 runs. The independent MCMC runs were combined for the better posterior effective sample size and tree. The starting time for the BBayA R. felis was set as 2,000 years BP and all other species were assumed as '0' years. The maximum likelihood tree was supplied as an initial tree for the Bayesian MCMC analysis. The coalescent constant prior and strict clock did not change the topology of the initial tree in the final output. A Burnin tree was produced after discarding the first 10% of trees generated. However, considering the dates obtained for most of the 126 genomes (based on publication date or isolation date, if available) used for evolutionary analyses, the dataset did not show temporal signals through the Tempest software (*i.e.*, negative correlations). The other alternative was to perform a date-randomization test. For this purpose, the BEAST analysis was run again considering the retrieved dates. The BEAUti file (.xml) necessary to run BEAST was processed with the tipdatingbeast R package (Rieux and Khatchikian, 2017) using the RandomDates function to generate 20 BEAUti (.xml) files like the original analysis but with the dates randomized of

the 127 sequences. The 20 BEAST analyses were run, and the results, along with the original analysis, were processed with the PlotDRT which makes a statistical analysis of the distribution of some resulting statistics from BEAST. In theory, these analyses should indicate 'An estimate of the substitution rate passes this test if its mean does not fall within the 95% credible intervals of rate estimates obtained using replicate data sets in which the sampling times have been randomized' (Duchene et al. 2015). These analyses however failed in every metric, showing that the DRT (date-randomization test) failed due to overlapping values between the original dataset and the replicates.

Supplementary Note 6 Recovering ancient pathogenic microbial taxa from human petrous bone

In contrast with the results reported by Margaryan et al. (2018), this study confirms that the DNA of ancient pathogenic microbial taxa can also be recovered from human petrous bone samples. Margaryan et al. (2018) initially focussed on the differential detection of a single pathogen, *Yersinia pestis*, from human teeth and petrous samples, subsequently showing a much higher microbial diversity in teeth than petrous bones, including various additional pathogenic and oral microbial taxa. The reasons cited for this result include the fact that the otic capsules of the petrous bones are harder than tooth roots, implying that very little exogenous DNA will penetrate into these bones. In our case, the remains analysed represented that of a child, the skull and teeth of which were still experiencing formative development and, therefore, not yet fully fused, developed and densified. In addition, chronic diseases and resulting comorbidities are associated with diminished bone mineral accrual and bone loss, and various paediatric disorders have been implicated in impaired bone health (Williams, 2016). It is therefore probable that the child displayed irregular and abnormally-low skeletal bone density and skeletal metabolism, in turn resulting in an increasing predisposition of pathogenic microbes to circulate through and enter the generally dense otic capsules of the petrous regions. This is supported by the documented *cribra orbitalia* in the child, a known indicator of childhood stress (Pfeiffer et al., 2019).

Besides the young age and compromised health of the child, taphonomic factors might further explain the differential preservation of microbial DNA in the petrous and tooth samples. The site from which the remains were retrieved comprised a mound formed by a shell midden overlooking the beach, ~ 46 m from the high-water mark. The damp saline conditions and loose sedimentary matrix may have resulted in increasingly rapid DNA degradation, particularly in the exposed sub-adult teeth (Latham and Miller, 2019). Ultimately, the sequencing strategy originally employed resulted in the sequencing of seven libraries derived from the left and right petrous samples, and only a single library from the upper premolar, introducing a significant bias in terms of the numbers of microbial reads recovered from the respective samples.

Supplementary Note 7 Pathogenicity and clinical symptoms of *Rickettsia felis* infection

Rickettsia felis, an insect-borne rickettsial pathogen and the causative agent of typhus-like flea-borne 'spotted fever', is an obligate intracellular bacterium in the order Rickettsiales (Angelakis et al., 2016). While cat- and dog-fleas (*Ctenocephalides felis* and *C. canis*) have been cited as the most probable vectors, >40 different haematophagous species of fleas, mosquitoes, ticks and mites have been identified as vectors (Legendre and Macaluso, 2017). As well as the identification of the African great apes (chimpanzees, gorillas, and bonobos) as vertebrate reservoirs responsible for the maintenance of *R. felis* in Africa, it has been proposed that humans are natural *R. felis* reservoirs (Mediannikov et al., 2014), just as they are for certain *Plasmodium* species (Gonçalves et al., 2017). *R. felis* is therefore capable of infecting multiple hosts and vectors, and co-feeding likely explains the enzootic spread of *R. felis* among variable host- and vector-populations (Angelakis et al., 2016; Brown and Macaluso, 2016). In addition, while rickettsial diseases are widely stated to represent emerging infectious pathogens, the historic influence of *Rickettsia* is well-known. Whereas the first evidence of *R. felis's* potential as a human pathogen surfaced in 1994 (Angelakis et al., 2016), the first reliable description of typhus-like disease appears in 1489 during the Spanish siege of Baza against the Moors during the War of Granada (1482 to 1492) (Pages et al., 2010). Ancient DNA analysis of human remains and body lice (*Pediculus humanus*) recovered from the graves of soldiers who perished during Napoleon's 1812 Russian Campaign, confirmed historic accounts of the presence of both trench fever (*Bartonella quintana*) and epidemic typhus (*Rickettsia prowazekii*) during the campaign (Raoult et al., 2006).

The clinical presentation of rickettsial diseases ranges from mild to severe. Without antibiotic treatment, murine or 'endemic' typhus, caused by *R. typhi*, exhibits a mortality rate of 4%, and Rocky Mountain spotted fever a mortality rate as high as 30% (Snowden and Bhimji, 2017). Epidemic typhus, caused by *R. prowazekii*, has a mortality rate which varies from 0.7% to 60% for untreated cases. Mortality rates as high as 66% has been reported for disease due to *R. rickettsii* occurring prior to 1920, preceding the discovery of antibiotics (Azad, 2007). The minimal genomic divergence distinguishing *R. felis* from other flea-associated strains suggests that it has the potential to be a human pathogen (Gillespie et al., 2015). The clinical manifestations of *R. felis* infection closely resemble those of flea-borne murine typhus (Blanto and Walker, 2016) which entails the abrupt onset of fever with accompanying headache, chills, myalgia, malaise and cutaneous maculopapular rashes (Angelakis et al., 2016; Legendre and Macaluso, 2017).

The similarity of typhus-like flea-borne rickettsioses symptoms to *R. typhi*, as well as the lack of specific diagnostics, has potentially resulted in the under-diagnosis of *R. felis* in many human cases (Legendre and Macaluso, 2017). In sub-Saharan Africa, *R. felis* is described as a common (~15 %) cause of illness among patients with 'fever of unknown origin', particularly in malaria-endemic regions (Brown and Macaluso, 2016). In some regions, the incidence of human *R. felis* infections far-

exceeds that of malaria. Diagnosis is problematic because symptoms are common to other infectious diseases, including mosquito-borne dengue fever (Flavivirus) and malaria (e.g., *P. falciparum*) and brucellosis (*B. melitensis*). *R. felis* has furthermore been detected in the blood and cerebrospinal fluid of those with an alternative and more compelling diagnosis, including malaria, cryptococcal meningitis and scrub typhus (Blanton and Walker, 2017). The clinical presentation of rickettsial diseases can vary from mild to very severe, with the case-fatality rate for highly virulent rickettsiae ranging from 2% to 30% (Azad, 2007). Human disease case fatality rates (CFRs), the proportion of patients that reportedly died as a result of infection, of 19% have been reported for untreated *R. felis* infections (Oliveira et al., 2002).

References

1. Angelakis, E. et al. *Rickettsia felis*: The complex journey of an emergent human pathogen. Trends Parasitol. **32**, doi: 10.1016/j.pt.2016.04.009 (2016).
2. Azad, A.A. Pathogenic Rickettsiae as bioterrorism agents. Ann N Y Acad Sci. **990**, 734-738. (2007).
3. Blanton, L.S. et al. Flea-borne Rickettsioses and Rickettsiae. Am J Trop Med Hyg. **96**, 53-56 (2017).
4. Bouckaert, R. et al. BEAST 2.5: An advanced software platform for Bayesian evolutionary analysis. PLoS Comput Biol. 15, doi: 10.1371/journal.pcbi.1006650 (2019).
5. Brown, L.D. et al. *Rickettsia felis*, an emerging flea-borne Rickettsiosis. Curr Trop Med Rep. **3**, 27-39 (2016).
6. Cunnington, A.J. The importance of pathogen load. PLoS Pathog. **11**, doi: 10.1371/journal.ppat.1004563 (2015).
7. Duchene, S. et al. The performance of the date-randomization test in phylogenetic analyses of time-structured virus data, Mol. Biol. Evol. **32**, 7, doi: https://doi.org/10.1093/molbev/msv056 (2015).
8. Dutour, O. Paleoparasitology and paleopathology: Synergies for reconstructing the past of human infectious diseases and their pathocenosis. Int J Paleopathol. **3**, 145-149 (2013).
9. Ferwerda, B. et al. TLR4 polymorphisms, infectious diseases, and evolutionary pressure during migration of modern humans. Proc Natl Acad Sci U S A. **104**, 16645-16650 (2007).
10. Gillespie, J.J. et al. Genomic diversification in strains of *Rickettsia felis* isolated from different arthropods. Genome Biol Evol. **7**, 35-56 (2015).
11. Gonçalves, B.P. et al. Examining the human infectious reservoir for *Plasmodium falciparum* malaria in areas of differing transmission intensity. Nat Commun. **26**, doi: 10.1038/s41467-017-01270-4. (2017).
12. Houldcroft, C.J. et al. Neanderthal genomics suggests a Pleistocene time frame for the first epidemiologic transition. Am J Phys Anthropol. **160**, 379-388 (2016).

13. Howes, R.E. et al. The global distribution of the Duffy blood group. Nat Commun. **266**, doi: 10.1038/ncomms1265. (2011).
14. Hublin, J.J. et al. New fossils from Jebel Irhoud, Morocco and the pan-African origin of *Homo sapiens*. Nature. **546**, doi: 10.1038/nature22336 (2017).
15. Ko, W. et al. Effects of natural selection and gene conversion on the evolution of human glycophorins coding for MNS blood polymorphisms in malaria-endemic African populations. Am J Hum Genet. **88**, doi: 10.1016/j.ajhg.2011.05.005 (2011).
16. Kwiatkowski, D.P. How malaria has affected the human genome and what human genetics can teach us about malaria. Am J Hum Genet. **77**, doi: 10.1086/432519. (2005).
17. Latham, K.E. et al. DNA recovery and analysis from skeletal material in modern forensic contexts. Forensic Sci Res. **4**, doi: 10.1080/20961790.2018.1515594 (2019).
18. Legendre, K.P. et al. *Rickettsia felis*: A review of transmission mechanisms of an emerging pathogen. Trop Med Infect Dis. 2, doi: 10.3390/tropicalmed2040064 (2017).
19. Lloyd-Smith, J.O. et al. Epidemic dynamics at the human-animal interface. Science **326**, 1362-1367 (2009).
20. Lombard, M. et al. South African and Lesotho Stone Age sequence updated (1). S Afr Archaeol Bull. **67**, 123-144 (2012).
21. Lopez, M. et al. Genomic evidence for local adaptation of hunter-gatherers to the African rainforest. Curr Biol. 29, doi: 10.1016/j.cub.2019.07.013 (2019).
22. Mann, A.E. et al. Differential preservation of endogenous human and microbial DNA in dental calculus and dentin. Sci Rep. **8**, doi: 10.1038/s41598-018-28091-9 (2018).
23. Margaryan, A. et al. Ancient pathogen DNA in human teeth and petrous bones. Ecol Evol. **8**, doi: 10.1002/ece3.3924 (2018).
24. McArdle, A.J. et al. When do co-infections matter? Curr Opin Infect Dis. **31**, doi: 10.1097/QCO.0000000000000447 (2018).
25. McManus, K.F. et al. Population genetic analysis of the DARC locus (Duffy) reveals adaptation from standing variation associated with malaria resistance in humans. PLoS Gen. **10**, doi. https://doi.org/10.1371/journal.pgen.1006560 (2017).
26. Mediannikov, O. et al. Common epidemiology of *Rickettsia felis* infection and malaria, Africa. Emerg Infect Dis. **19**, doi: 10.3201/eid1911.130361 (2014).
27. Moodley, Y. et al. Age of the association between *Helicobacter pylori* and man. PLoS Pathog. **8**, doi: 10.1371/journal.ppat.1002693. (2012).
28. Oliveira, R.P. et al. *Rickettsia felis* in *Ctenocephalides* spp. fleas, Brazil. Emerg Infect Dis. **8**, doi: https://dx.doi.org/10.3201/eid0803.010301 (2002).
29. Ollomo, B. et al. A new malaria agent in African hominids. PLoS Pathog. **5**, doi: 10.1371/journal.ppat.1000446 (2009).

30. Pages, F. et al. The past and present threat of vector-borne diseases in deployed troops. Clin Microbiol Infect. **16**, 209-24 (2010).
31. Pfeiffer, S. et al. The people behind the samples: Biographical features of past hunter-gatherers from KwaZulu-Natal who yielded aDNA. Int J Paleopathol. **24**, 158-164 (2019).
32. Raoult, D. et al. Evidence for louse-transmitted diseases in soldiers of Napoleon's Grand Army in Vilnius. J Infect Dis. **193**, 112-120 (2006).
33. Rieux, S. et al. tipdatingbeast: an r package to assist the implementation of phylogenetic tip-dating tests using beast. Mol. Ecol. Resour. **17**, doi: 10.1111/1755-0998.12603 (2017)
34. Schlebusch, C.M. et al. Southern African ancient genomes estimate modern human divergence to 350,000 to 260,000 years ago. Science. **358**, 652-655 (2017).
35. Silva, J.C. et al. Genome sequences reveal divergence times of malaria parasite lineages. Parasitology **138**, 1737-1749 (2011).
36. Snowden, J. et al. *Rickettsia* rickettsiae (Rocky Mountain Spotted Fever). StatPearls Publishing, available from https://www.ncbi.nlm.nih.gov/books/NBK430881/ (2017).
37. Tanabe, K. et al. *Plasmodium falciparum* accompanied the human expansion out of Africa. Curr Biol. **20**, 1283-1289 (2010).
38. Taylor, L.H. et al. Risk factors for human disease emergence. Philos Trans R Soc Lond Ser B Biol Sci. **356**, 983-989 (2001).
39. Thepparit, C. et al. Isolation of a rickettsial pathogen from a non-hematophagous arthropod. PLoS One. **6**, doi: 10.1371/journal.pone.0016396 (2011).
40. Wang, X. et al. Specific inactivation of two immunomodulatory SIGLEC genes during human evolution. Proc Natl Acad Sci U S A. **109**, doi: 10.1073/pnas.1119459109. (2012).
41. Wardeh, M. et al. Database of host-pathogen and related species interactions, and their global distribution. Sci Data **2**, doi: 10.1038/sdata.2015.49 (2015).
42. Williams, K.M. Update on bone health in paediatric chronic disease. Endocrinol Metab Clin North Am. **45**, doi: 10.1016/j.ecl.2016.01.009 (2016).
43. Wolfe, N.D. et al. Origins of major human infectious diseases. Nature **447**, 279-283 (2007).
44. Woolhouse, M.E.L. et al. Host range and emerging and re-emerging pathogens. Emerg Infect Dis. **11**, 1842-1847 (2005).

Supplementary Figures

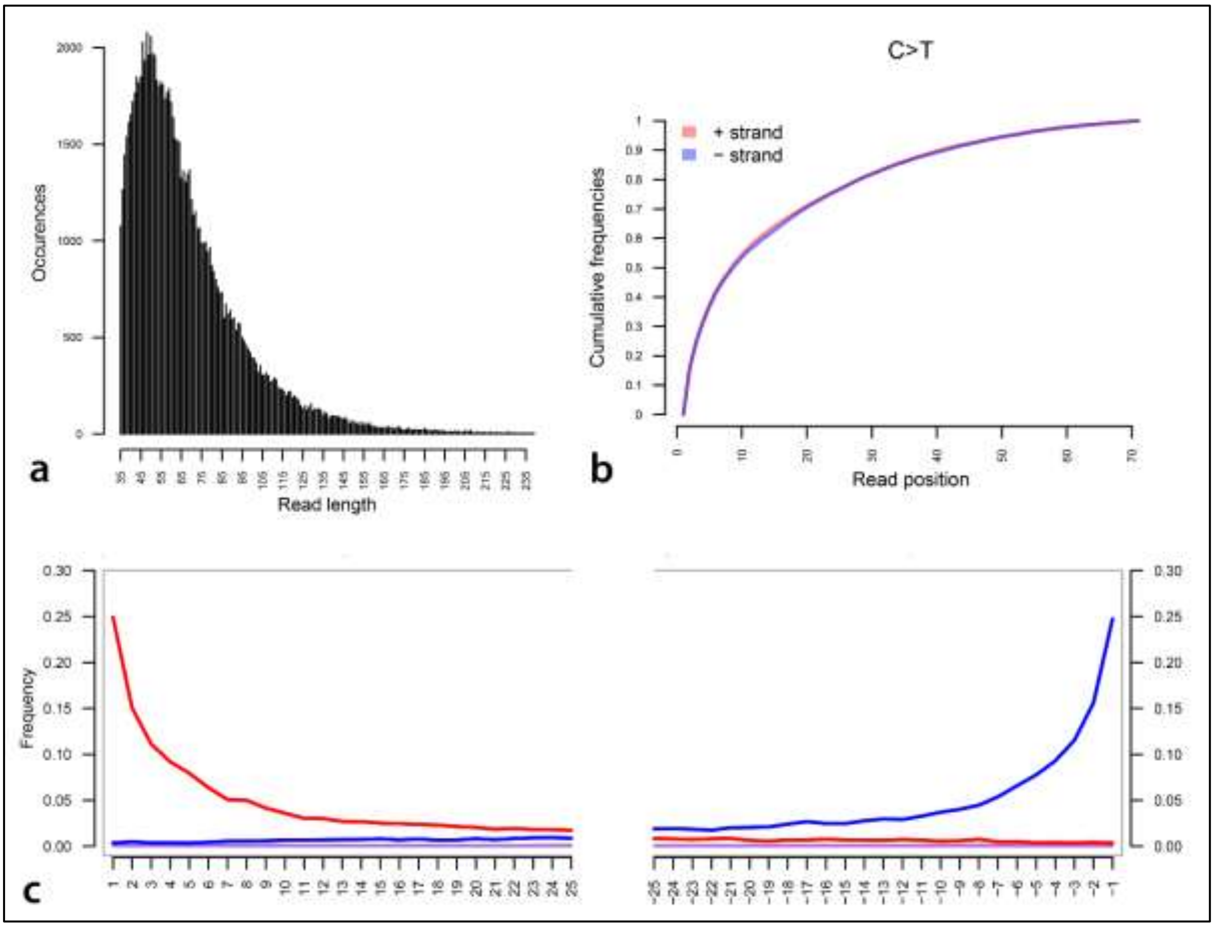

Figure S1. Damage pattern and read-length distribution analysis of the human host's (BBayA) DNA exhibit a similar DNA damage profile and short (*i.e.*, damaged) read-length distribution to that of the *R. felis* DNA sequence reads analysed. **a**) DNA fragment read-length distributions of the BBayA host reads, **b**) C-T read strand positions and **c**) G-to-A and C-to-T misincorporations are plotted in blue and red, respectively, and the grey lines indicate all possible misincorporations.

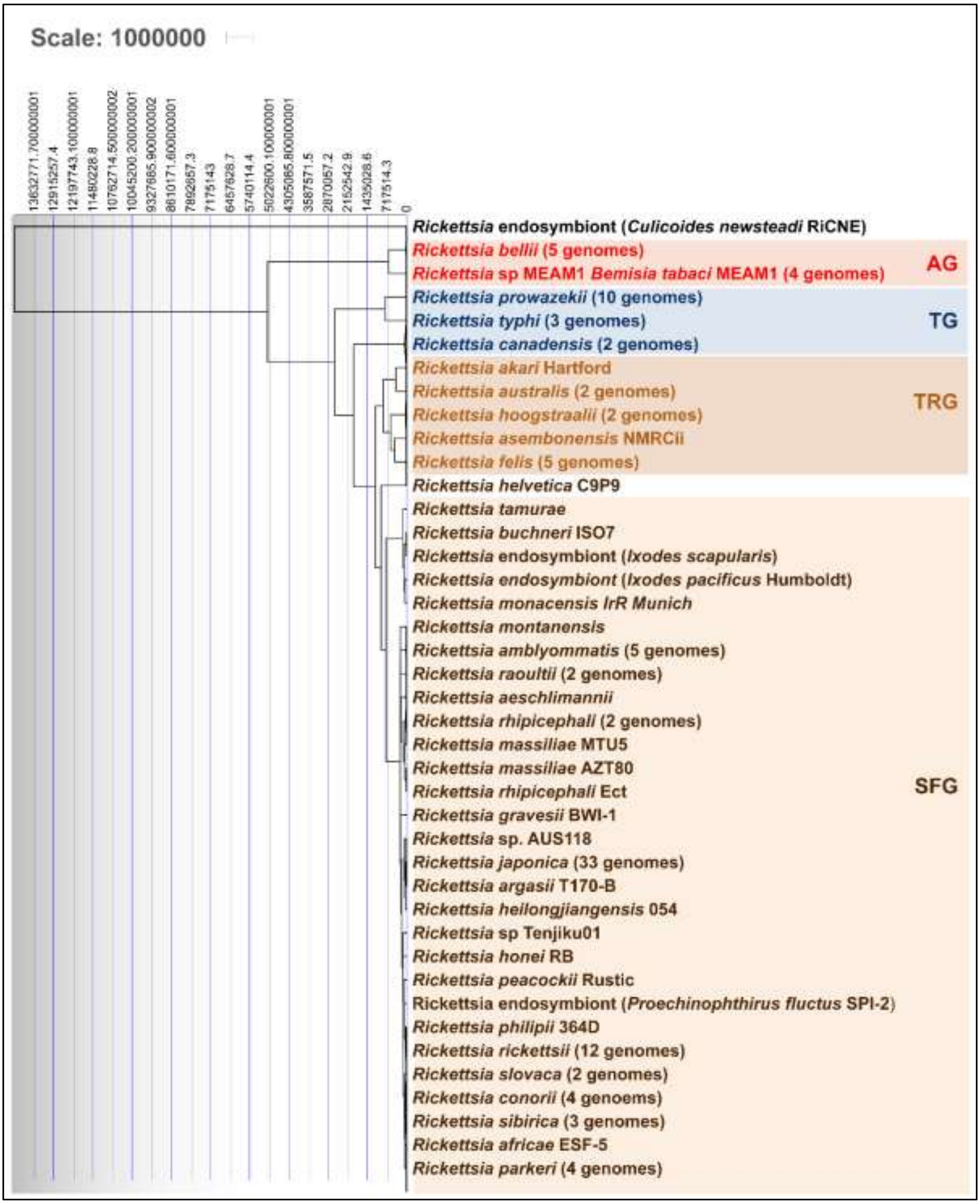

Figure S2. Molecular clock and divergence time analysis of the ancient BbayA *R. felis* genome was performed using all (*i.e.*, 126) currently available NCBI reference genomes. The MCMC algorithm was applied on the codon alignment of 138 core genes using a strict clock, a coalescent constant and the GTRGI substitution model in Beast2.

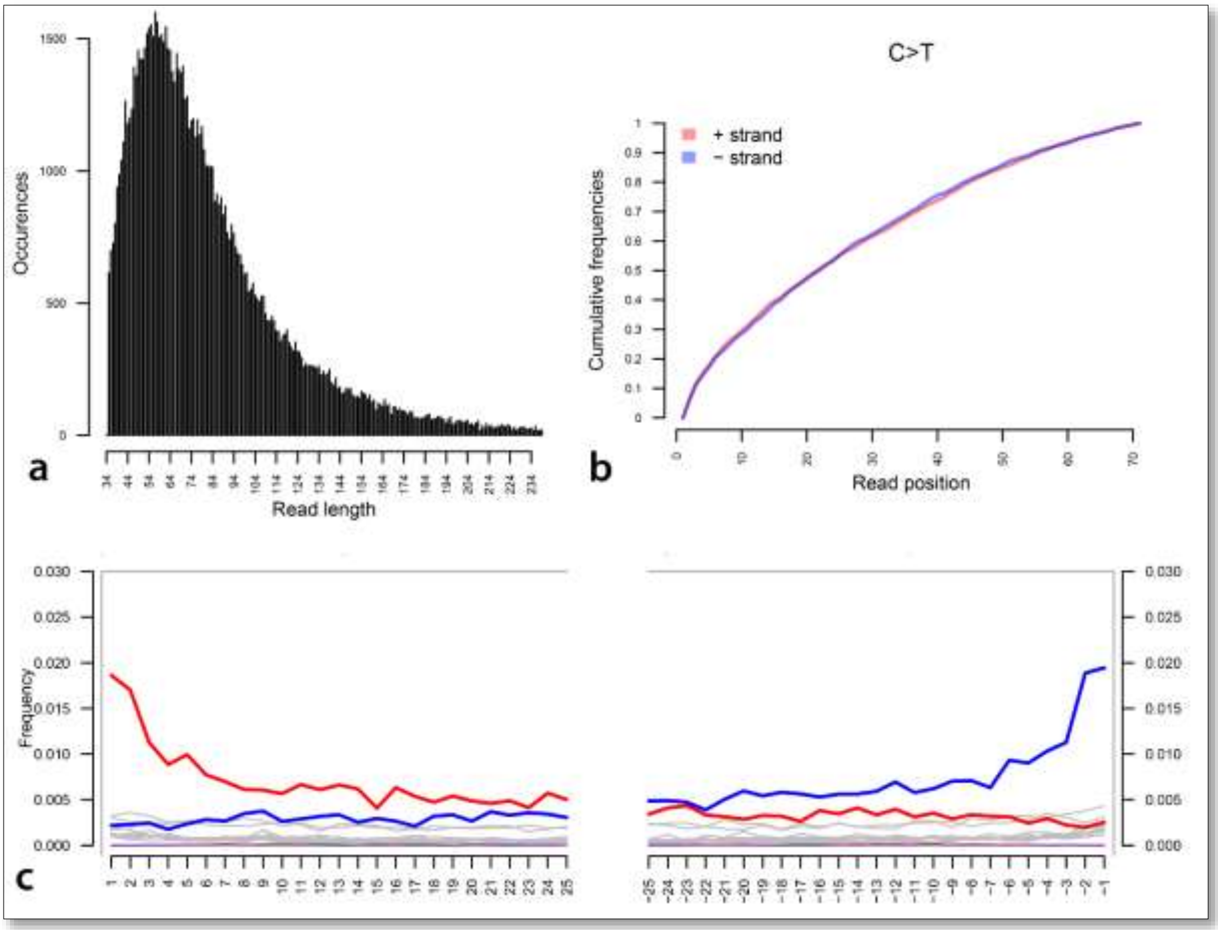

Figure S3. DNA damage pattern analysis for the BBayA *R. felis* reads using mapDamage with AdapaterRemoval 2.3.1 (https://github.com/MikkelSchubert/adapterremoval/issues/32#issuecomment-504758137) and selecting the option '--preserve5p' resulted in a DNA damage plot comparable to that shown in Fig. 2 b. **a**) DNA fragment read-length distributions of the BBayA *R. felis* reads, **b**) C-T read strand positions and **c**) G-to-A and C-to-T misincorporations are plotted in blue and red, respectively, and the grey lines indicate all possible misincorporations.

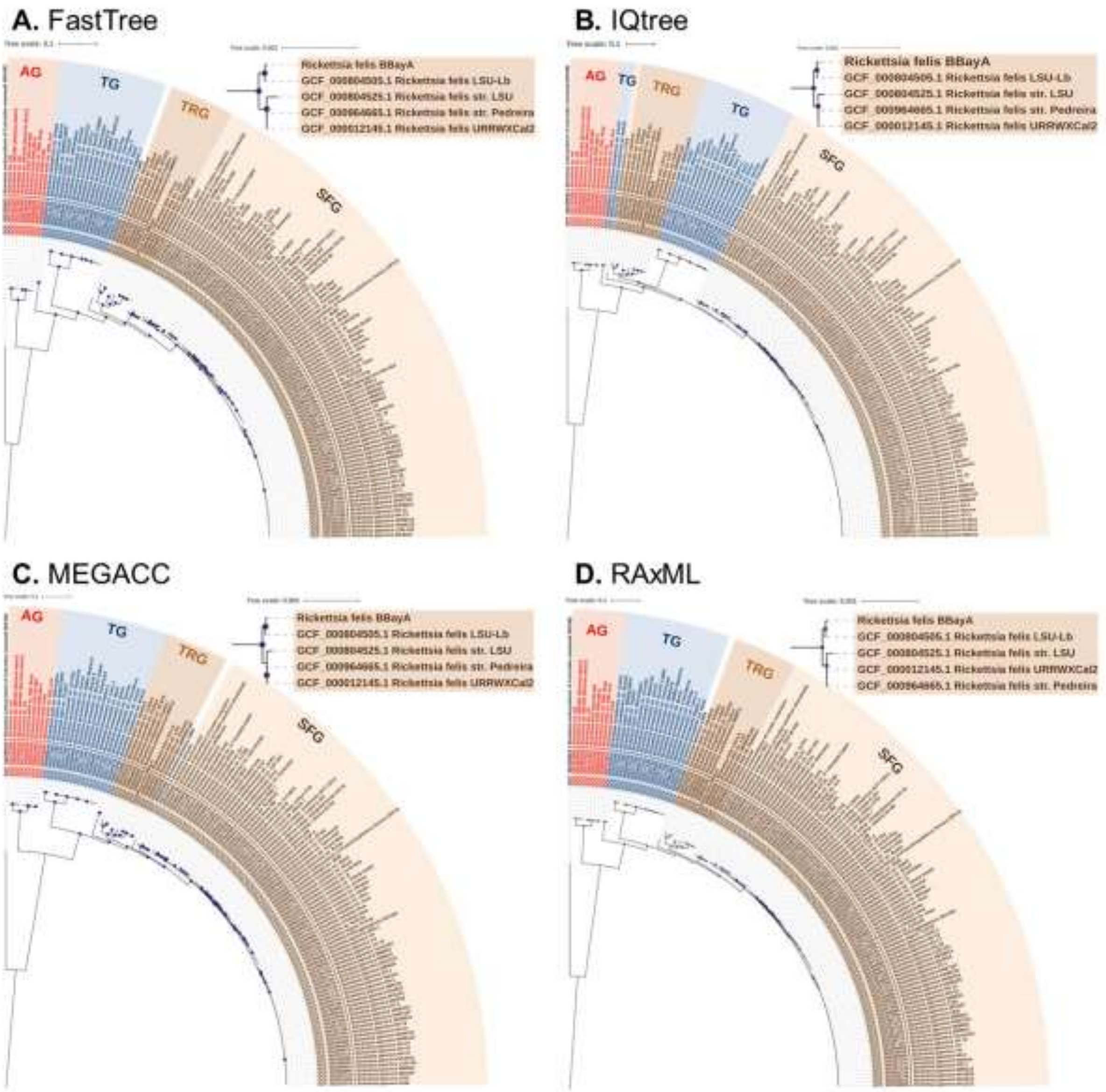

E.

Tree	logL	deltaL	bp-RELL		p-KH		p-SH		p-WKH		p-WSH		c-ELW		p-AU	
FastTree	-585759.149	26.819	0.153	+	0.287	+	0.498	+	0.287	+	0.611	+	0.151	+	0.337	+
IQtree	-585732.33	0	0.708	+	0.713	+	1	+	0.713	+	0.838	+	0.706	+	0.727	+
MEGACC	-586138.939	406.61	0	-	0	-	0	-	0	-	0	-	3.24E-92	-	0.000127	-
RAxML	-585759.13	26.8	0.139	+	0.287	+	0.499	+	0.287	+	0.641	+	0.143	+	0.315	+

Plus signs denote the 95% confidence sets.
Minus signs denote significant exclusion.
All tests performed 10000 resamplings using the RELL method.

Figure S4. Additional maximum-likelihood trees constructed using the same codon alignment with the FastTree version 2.1.10 with -gtr and -gamma options, the RAxML version 8.2.11 with -m GTRGAMMA, -#100 (to search the best tree between 100 replicates) and -# autoMR option to determine node bootstraps with automatic number of replicates and MEGA-CC version 11.0.10 using

GTR (G+I) model and bootstrap support of 100 replicates. These phylogenomic reconstructions were compared using the approximately unbiased (AU) test implemented in IQ-TREE v.1.5.5 with the options -n 0 -zb 10000 -au -zw. The *p*-values for the AU test of the FastTree (*p*-value 0.337), IQtree (*p*-value 0.727) and RAxML (*p*-value 0.315) reconstructions indicated these trees as 95 % confident sets, while the MEGA-CC tree got a significant exclusion (*p*-value 0.000127).

Supplementary Data 1-6

Supplementary Data 1. Raw taxonomic reads derived from the Kraken analyses for the left petrous bone (LPB), right petrous bone (RPB) and the upper left premolar (ULPM).

Supplementary Data 2. Mapping of the BBayA aDNA sequence dataset was performed on a competitive basis against bacterial and parasitic genomes, and a complete human genome. NCBI reference assembly genomes are indicated for all the authenticated taxa detected in the BBayA metagenomic dataset.

Supplementary Data 3. The 126 NCBI reference genomes initially used to identify the closest genomic homologues to the ancient BBayA *R. felis* strain.

Supplementary Data 4 a, b, c, d, e. The NCBI reference genomes used for phylogenetic analyses and comparison of the BBayA *R. felis* to *R. felis* LSU-Lb, *R. felis* URRWxCal2, *R. typhi*, *R. prowazekii* and *R. africae*.

Supplementary Data 5 a, b. Information concerning the quality analyses of all analysed Rickettsia reference genomes as evaluated with the CheckM v1.1.3 software package.

Supplementary Data 6. Phylogenomic reconstructions were compared using the approximately unbiased (AU) test implemented in IQ-TREE v.1.5.5 with the options -n 0 -zb 10000 -au -zw. The *p*-values for the AU test of the FastTree (*p*-value 0.337), IQtree (*p*-value 0.727) and RAxML (*p*-value 0.315) reconstructions indicated these trees as 95 % confident sets, while the MEGA-CC tree got a significant exclusion (*p*-value 0.000127).
