# Supplementary material for: Rickettsia felis DNA recovered from a child who lived in southern Africa 2000 years ago
Source: Commun Biol. 2023 Mar 3;6:240. doi: 10.1038/s42003-023-04582-y (PMC9984395; doi:10.1038/s42003-023-04582-y)
Supplement: Supplementary file 3 — Description of Additional Supplementary Files [file 42003_2023_4582_MOESM3_ESM.pdf]

Description of Additional Supplementary Files

File name: Supplementary Data 1
Description: Raw taxonomic reads derived from the Kraken analyses for the left petrous bone (LPB), right petrous bone (RPB) and the upper left premolar (ULPM).

File name: Supplementary Data 2
Description: Mapping of the BBayA aDNA sequence dataset was performed on a competitive basis against bacterial and parasitic genomes, and a complete human genome. NCBI reference assembly genomes are indicated for all the authenticated taxa detected in the BBayA metagenomic dataset.

File name: Supplementary Data 3
Description: The 126 NCBI reference genomes initially used to identify the closest genomic homologues to the ancient BBayA R. felis strain.

File name: Supplementary Data 4
Description: a, b, c, d, e. The NCBI reference genomes used for phylogenetic analyses and comparison of the BBayA R. felis to R. felis LSU-Lb, R. felis URRWxCal2, R. typhi, R. prowazekii and R. africae.

File name: Supplementary Data 5
Description: a, b. Information concerning the quality analyses of all analysed Rickettsia reference genomes as evaluated with the CheckM v1.1.3 software package.

File name: Supplementary Data 6
Description: Phylogenomic reconstructions were compared using the approximately unbiased (AU) test implemented in IQ-TREE v.1.5.5 with the options -n 0 -zb 10000 -au -zw. The p-values for the AU test of the FastTree (p-value 0.337), IQtree (p-value 0.727) and RAxML (p-value 0.315) reconstructions indicated these trees as 95 % confident sets, while the MEGA-CC tree got a significant exclusion (p-value 0.000127).
