# Supplementary material for: Rickettsia felis DNA recovered from a child who lived in southern Africa 2000 years ago
Source: Commun Biol. 2023 Mar 3;6:240. doi: 10.1038/s42003-023-04582-y (PMC9984395; doi:10.1038/s42003-023-04582-y)
Supplement: Supplementary file 5 — Reporting Summary [file 42003_2023_4582_MOESM5_ESM.pdf]

nature research

Corresponding author(s): Riaan F. Rifkin
Marlize Lombard

Last updated by author(s): 9 February 2023

Reporting Summary

Nature Research wishes to improve the reproducibility of the work that we publish. This form provides structure for consistency and transparency in reporting. For further information on Nature Research policies, see our Editorial Policies Editorial Policy Checklist

Statistics

For all statistical analyses, confirm that the following items are present in the figure legend, table legend, main text, or Methods section.

n/a	Confirmed	
☐	☒	The exact sample size (n
☒	☐	A statement on whether measurements were taken from distinct samples or whether the same sample was measured repeatedly
☐	☒	The statistical test(s) used AND whether they are one- or two-sided<br>Only common tests should be described solely by name; describe more complex techniques in the Methods section.
☒	☐	A description of all covariates tested
☒	☐	A description of any assumptions or corrections, such as tests of normality and adjustment for multiple comparisons
☒	☐	A full description of the statistical parameters including central tendency (e.g. means) or other basic estimates (e.g. regression coefficient) AND variation (e.g. standard deviation) or associated estimates of uncertainty (e.g. confidence intervals)
☒	☐	For null hypothesis testing, the test statistic (e.g. F t r P<br>Give P values as exact values whenever suitable.
☐	☒	For Bayesian analysis, information on the choice of priors and Markov chain Monte Carlo settings
☒	☐	For hierarchical and complex designs, identification of the appropriate level for tests and full reporting of outcomes
☒	☐	Estimates of effect sizes (e.g. Cohen's d r

Our web collection on statistics for biologists

Software and code

Policy information about availability of computer code

Data collection	This work is based on the analyses of a shotgun metagenome dataset generated as per a stuty published by Schlebusch et al in 2017, titled 'Southern African ancient genomes estimate modern human divergence to 350,000 to 260,000 years ago' (see https://science.sciencemag.org/content/358/6363/652.abstract?casa_token=MlaeaAuLj30AAAAA:ANTc9ayhtRKvKArH2ZyPX9h6QxT1A6HSI1l_9Ee9WdN9GDNUey8vmQIaTE0OYp96GWzvpZDEvxwlnbij).
Data analysis	We have provided a full data availability statement in the Manuscript and we provide all the references for all the publically-available and widely used software used in this work.

For manuscripts utilizing custom algorithms or software that are central to the research but not yet described in published literature, software must be made available to editors and reviewers. We strongly encourage code deposition in a community repository (e.g. GitHub). See the Nature Research guidelines for submitting code & software

Data

Policy information about availability of data

All manuscripts must include a data availability statement

- Accession codes, unique identifiers, or web links for publicly available datasets
- A list of figures that have associated raw data
- A description of any restrictions on data availability

We have provided a full data availability statement in the Manuscript and we provide all references for all the software used in this work. Accession codes including web-links for all the processed datasets will be made available before publication. Reviewers may view these files should they request to do so. Supplementary Figures and Tables are provided as referred to in the Manuscript text. No restrictions will be placed concerning the availability of data generated in this work.

Field-specific reporting

Please select the one below that is the best fit for your research. If you are not sure, read the appropriate sections before making your selection.

☐ Life sciences ☐ Behavioural & social sciences ☒ Ecological, evolutionary & environmental sciences

For a reference copy of the document with all sections, see nature.com/documents/nr-reporting-summary-flat.pdf

Life sciences study design

All studies must disclose on these points even when the disclosure is negative.

Sample size	Disclosed in the manuscript.
Data exclusions	Not applicable.
Replication	Not applicable.
Randomization	Not applicable.
Blinding	Not applicable.

Behavioural & social sciences study design

All studies must disclose on these points even when the disclosure is negative.

Study description	Briefly describe the study type including whether data are quantitative, qualitative, or mixed-methods (e.g. qualitative cross-sectional, quantitative experimental, mixed-methods case study).
Research sample	State the research sample (e.g. Harvard university undergraduates, villagers in rural India) and provide relevant demographic information (e.g. age, sex) and indicate whether the sample is representative. Provide a rationale for the study sample chosen. For studies involving existing datasets, please describe the dataset and source.
Sampling strategy	Describe the sampling procedure (e.g. random, snowball, stratified, convenience). Describe the statistical methods that were used to predetermine sample size OR if no sample-size calculation was performed, describe how sample sizes were chosen and provide a rationale for why these sample sizes are sufficient. For qualitative data, please indicate whether data saturation was considered, and what criteria were used to decide that no further sampling was needed.
Data collection	Provide details about the data collection procedure, including the instruments or devices used to record the data (e.g. pen and paper, computer, eye tracker, video or audio equipment) whether anyone was present besides the participant(s) and the researcher, and whether the researcher was blind to experimental condition and/or the study hypothesis during data collection.
Timing	Indicate the start and stop dates of data collection. If there is a gap between collection periods, state the dates for each sample cohort.
Data exclusions	If no data were excluded from the analyses, state so OR if data were excluded, provide the exact number of exclusions and the rationale behind them, indicating whether exclusion criteria were pre-established.
Non-participation	State how many participants dropped out/declined participation and the reason(s) given OR provide response rate OR state that no participants dropped out/declined participation.
Randomization	If participants were not allocated into experimental groups, state so OR describe how participants were allocated to groups, and if allocation was not random, describe how covariates were controlled.

Ecological, evolutionary & environmental sciences study design

All studies must disclose on these points even when the disclosure is negative.

Study description	Information is provided in the Manuscript.
Research sample	Provided in the Manuscript.
Sampling strategy	All the samples (i.e., teeth and petrous samples) from which DNA was extracted and subsequently sequenced provided genome data which was used (processed) in this work.

Data collection	*Information concerning the source and Museum curation of the human skeletal materials from which the genomicdata is derived is provided in the Manuscript.*
Timing and spatial scale	*Not applicable.*
Data exclusions	*Data processing methods are described in the Materials and Methods section, including information concerning the validation of the antiquity of ancient DNA reads, which, in turn would have resulted in the exclusion of microbial taxa should these not have been confirmed as truly ancient.*
Reproducibility	*Not applicable.*
Randomization	*Describe how samples/organisms/participants were allocated into groups. If allocation was not random, describe how covariates were controlled. If this is not relevant to your study, explain why.*
Blinding	*Not applicable.*

Did the study involve field work? ☐ Yes ☒ No

Field work, collection and transport

Field conditions	*Not applicable.*
Location	*The original sample site (i.e., the archaeological context) is described and the approximate location is provided.*
Access & import/export	*Not applicable.*
Disturbance	*Not applicable.*

Reporting for specific materials, systems and methods

We require information from authors about some types of materials, experimental systems and methods used in many studies. Here, indicate whether each material, system or method listed is relevant to your study. If you are not sure if a list item applies to your research, read the appropriate section before selecting a response.

Materials & experimental systems

n/a	Involved in the study	
☒	☐	Antibodies
☒	☐	Eukaryotic cell lines
☐	☒	Palaeontology and archaeology
☒	☐	Animals and other organisms
☒	☐	Human research participants
☒	☐	Clinical data
☒	☐	Dual use research of concern

Methods

n/a	Involved in the study	
☒	☐	ChIP-seq
☒	☐	Flow cytometry
☒	☐	MRI-based neuroimaging

Antibodies

Antibodies used	*Describe all antibodies used in the study; as applicable, provide supplier name, catalog number, clone name, and lot number.*
Validation	*Describe the validation of each primary antibody for the species and application, noting any validation statements on the manufacturer's website, relevant citations, antibody profiles in online databases, or data provided in the manuscript.*

Eukaryotic cell lines

Policy information about cell lines

Cell line source(s)	*State the source of each cell line used.*
Authentication	*Describe the authentication procedures for each cell line used OR declare that none of the cell lines used were authenticated.*
Mycoplasma contamination	*Confirm that all cell lines tested negative for mycoplasma contamination OR describe the results of the testing for mycoplasma contamination OR declare that the cell lines were not tested for mycoplasma contamination.*
Commonly misidentified lines (See ICLAC	*Name any commonly misidentified cell lines used in the study and provide a rationale for their use.*

Palaeontology and Archaeology

Specimen provenance	All information is provided in the Manuscript and in the Supplementary Materials sections.
Specimen deposition	All information is provided in the Manuscript and in the Supplementary Materials sections.
Dating methods	Described in the Manuscript and in the Supplementary Materials sections.

☒ Tick this box to confirm that the raw and calibrated dates are available in the paper or in Supplementary Information.

Ethics oversight	All approvals and permission have been met.

Note that full information on the approval of the study protocol must also be provided in the manuscript.

Animals and other organisms

Policy information about studies involving animals; ARRIVE guidelines

Laboratory animals	For laboratory animals, report species, strain, sex and age OR state that the study did not involve laboratory animals.
Wild animals	Provide details on animals observed in or captured in the field; report species, sex and age where possible. Describe how animals were caught and transported and what happened to captive animals after the study (if killed, explain why and describe method; if released, say where and when) OR state that the study did not involve wild animals.
Field-collected samples	For laboratory work with field-collected samples, describe all relevant parameters such as housing, maintenance, temperature, photoperiod and end-of-experiment protocol OR state that the study did not involve samples collected from the field.
Ethics oversight	Identify the organization(s) that approved or provided guidance on the study protocol, OR state that no ethical approval or guidance was required and explain why not.

Note that full information on the approval of the study protocol must also be provided in the manuscript.

Human research participants

Policy information about studies involving human research participants

Population characteristics	Describe the covariate-relevant population characteristics of the human research participants (e.g. age, gender, genotypic information, past and current diagnosis and treatment categories). If you filled out the behavioural & social sciences study design questions and have nothing to add here, write "See above."
Recruitment	Describe how participants were recruited. Outline any potential self-selection bias or other biases that may be present and how these are likely to impact results.
Ethics oversight	1

Note that full information on the approval of the study protocol must also be provided in the manuscript.

Clinical data

Policy information about clinical studies

All manuscripts should comply with the ICMJE guidelines for publication of clinical research and must include a completed CONSORT checklist

Clinical trial registration	Provide the trial registration number from ClinicalTrials.gov or an equivalent agency.
Study protocol	Note where the full trial protocol can be accessed OR if not available, explain why.
Data collection	Describe the settings and locales of data collection, noting the time periods of recruitment and data collection.
Outcomes	1

Dual use research of concern

Policy information about dual use research of concern

Hazards

Could the accidental, deliberate or reckless misuse of agents or technologies generated in the work, or the application of information presented in the manuscript, pose a threat to:

No	Yes	
☒	☐	Public health
☒	☐	National security
☒	☐	Crops and/or livestock
☒	☐	Ecosystems
☒	☐	Any other significant area

Experiments of concern

Does the work involve any of these experiments of concern:

No	Yes	
☒	☐	Demonstrate how to render a vaccine ineffective
☒	☐	Confer resistance to therapeutically useful antibiotics or antiviral agents
☒	☐	Enhance the virulence of a pathogen or render a nonpathogen virulent
☒	☐	Increase transmissibility of a pathogen
☒	☐	Alter the host range of a pathogen
☒	☐	Enable evasion of diagnostic/detection modalities
☒	☐	Enable the weaponization of a biological agent or toxin
☒	☐	Any other potentially harmful combination of experiments and agents

ChIP-seq

Data deposition

☐ Confirm that both raw and final processed data have been deposited in a public database such as GEO

☐ Confirm that you have deposited or provided access to graph files (e.g. BED files) for the called peaks.

Data access links<br>May remain private before publication.	For "Initial submission" or "Revised version" documents, provide reviewer access links. For your "Final submission" document, provide a link to the deposited data.
Files in database submission	Provide a list of all files available in the database submission.
Genome browser session<br>(e.g. UCSC	Provide a link to an anonymized genome browser session for "Initial submission" and "Revised version" documents only, to enable peer review. Write "no longer applicable" for "Final submission" documents.

Methodology

Replicates	Describe the experimental replicates, specifying number, type and replicate agreement.
Sequencing depth	Describe the sequencing depth for each experiment, providing the total number of reads, uniquely mapped reads, length of reads and whether they were paired- or single-end.
Antibodies	Describe the antibodies used for the ChIP-seq experiments; as applicable, provide supplier name, catalog number, clone name, and lot number.
Peak calling parameters	Specify the command line program and parameters used for read mapping and peak calling, including the ChIP, control and index files used.
Data quality	Describe the methods used to ensure data quality in full detail, including how many peaks are at FDR 5% and above 5-fold enrichment.
Software	Describe the software used to collect and analyze the ChIP-seq data. For custom code that has been deposited into a community repository, provide accession details.

Flow Cytometry

Plots

Confirm that:

- [ ] The axis labels state the marker and fluorochrome used (e.g. CD4-FITC).
- [ ] The axis scales are clearly visible. Include numbers along axes only for bottom left plot of group (a 'group' is an analysis of identical markers).
- [ ] All plots are contour plots with outliers or pseudocolor plots.
- [ ] A numerical value for number of cells or percentage (with statistics) is provided.

Methodology

Sample preparation	Describe the sample preparation, detailing the biological source of the cells and any tissue processing steps used.
Instrument	Identify the instrument used for data collection, specifying make and model number.
Software	Describe the software used to collect and analyze the flow cytometry data. For custom code that has been deposited into a community repository, provide accession details.
Cell population abundance	Describe the abundance of the relevant cell populations within post-sort fractions, providing details on the purity of the samples and how it was determined.
Gating strategy	Describe the gating strategy used for all relevant experiments, specifying the preliminary FSC/SSC gates of the starting cell population, indicating where boundaries between "positive" and "negative" staining cell populations are defined.

- [ ] Tick this box to confirm that a figure exemplifying the gating strategy is provided in the Supplementary Information.

Magnetic resonance imaging

Experimental design

Design type	Indicate task or resting state; event-related or block design.
Design specifications	Specify the number of blocks, trials or experimental units per session and/or subject, and specify the length of each trial or block (if trials are blocked) and interval between trials.
Behavioral performance measures	State number and/or type of variables recorded (e.g. correct button press, response time) and what statistics were used to establish that the subjects were performing the task as expected (e.g. mean, range, and/or standard deviation across subjects).

Acquisition

Imaging type(s)	Specify: functional, structural, diffusion, perfusion.
Field strength	Specify in Tesla
Sequence & imaging parameters	Specify the pulse sequence type (gradient echo, spin echo, etc.), imaging type (EPI, spiral, etc.), field of view, matrix size, slice thickness, orientation and TE/TR/flip angle.
Area of acquisition	1

Diffusion MRI ☐ Used ☒ Not used

Preprocessing

Preprocessing software	Provide detail on software version and revision number and on specific parameters (model/functions, brain extraction, segmentation, smoothing kernel size, etc.).
Normalization	If data were normalized/standardized, describe the approach(es): specify linear or non-linear and define image types used for transformation OR indicate that data were not normalized and explain rationale for lack of normalization.
Normalization template	1
Noise and artifact removal	1
Volume censoring	1

Statistical modeling & inference

Model type and settings	Specify type (mass univariate, multivariate, RSA, predictive, etc.) and describe essential details of the model at the first and second levels (e.g. fixed, random or mixed effects; drift or auto-correlation).
Effect(s) tested	Define precise effect in terms of the task or stimulus conditions instead of psychological concepts and indicate whether ANOVA or factorial designs were used.

Specify type of analysis: ☐ Whole brain ☐ ROI-based ☐ Both

Statistic type for inference (See Eklund et al. 2016	Specify voxel-wise or cluster-wise and report all relevant parameters for cluster-wise methods.
Correction	Describe the type of correction and how it is obtained for multiple comparisons (e.g. FWE, FDR, permutation or Monte Carlo).

Models & analysis

n/a	Involved in the study
☐	☐ Functional and/or effective connectivity
☐	☐ Graph analysis
☒	☐ Multivariate modeling or predictive analysis

Functional and/or effective connectivity	1
Graph analysis	Report the dependent variable and connectivity measure, specifying weighted graph or binarized graph, subject- or group-level, and the global and/or node summaries used (e.g. clustering coefficient, efficiency, etc.).
Multivariate modeling and predictive analysis	Specify independent variables, features extraction and dimension reduction, model, training and evaluation metrics.
